# Medical Literature

**Published:** 1853-09

**Authors:** Freeman Knowles


### *The duty of Physicians to sustain our Periodical Literature.*

BY FREEMAN KNOWLES, M. D.

There are perhaps few subjects of more importance to the profession at large, than that of our periodical medical literature; nor is there one in which the great mass of the profession are so remiss in furnishing the "material aid" for the support of a good medical journal, and facts and discoveries on which it must subsist.

It has become proverbially true, that many of our best and most experienced physicians never furnish a line or a word of encouragement to the conductors of our medical journals; while here in the West, is it too much to say that not over half even subscribe for and take any medical periodical whatever.

Brethren, these things should not be so. If you wish to hold an honorable rank in your profession and contribute your full share to make that profession to which you are devoting your lives, what it should be—an honor to yourselves and a blessing to mankind—support your periodical press, support it by words of lofty cheer, support it by subscribing for home as well as foreign journals, and, lastly, though not least, support it by your contribution of facts and experiments, and you will soon find that such labor is twice blessed—that it blesses both giver and receiver.

Young men who have closed their period of pupilage with their preceptor, finished their last course of lectures at their alma mater, received their diploma with the broad seal of the institution, are too apt to think they have closed their medical education, and that thenceforward they have nothing to do but wait quietly for professional distinction and emolument. To all such let me remark, you have mistaken your vocation; professional drones have been the canker and blight on all true professional distinction; and, though in such a course a man may obtain his bread, he never will rise above a degrading mediocrity in his cause, and will be rather a curse than a blessing to the people among whom he may be situated. Such a course, let me remark, is not in accordance with your duty to yourselves, your fellow men, your country and your God; for be it remembered, that progress is the great law of our being; and when you have left the office of your preceptor and the halls of your alma mater, resolve to be a student of nature, the wide arcana is open before you and if you are true to her and yourself, you may enter into her temple and feast upon her transcendant mysteries.

What then, let me ask, has produced the distinction of the Bells, the Hunters, the Beaches and Gregorys, of England; the Dupeytrans, Cuviers and Bichats, of France, with a host of other distinguished names all over the continent. And again, what has enabled so many of our own distinguished sons to scale the rugged paths of eminence, and with their own hand to write immortality upon the Temple of Fame. It has been industry, energy, perseverance, and an indomitable will and determination to succeed. All should remember that Minerva is a jealous goddess, and puts a high price upon the honors of her court, and that she will not permit her pupils to waste their hours in soft slumbers and yet obtain the rewards of merit,—it is only on the heights of Parnassus that she distributes her laurels. We must therefore climb, and cling to its rocks and precipices, its boulders and evergreens, till we gain the summit, before we can wear the crown.

How important, then, to professional usefulness and distinction, are habits of industry and untiring perseverance in the cultivation of medical science. All around us the great fields of vegetable, mineral and animal creation are inviting scrutiny and investigation. There is labor enough for each and enough for all, and when we enter this field we should enter it as free and independent observers, with no theories to bolster, no prejudices to gratify and no opinions to sustain, but ever remember that *skepticism in medicine is no crime.* We shall then in all our investigations be guided by an honest desire after truth. Thus truth will become our motto, and in the same ratio that we seek after truth with energy, industry and perseverance, shall we find it.

We wish to call the attention of the profession to another important consideration, on the success of which much of our character and medical literature depends, and that is the support of our HOME MEDICAL SCHOOLS.

We are fully aware that much may be said in favor of our excellent Eastern Medical Institutions. It is also true that very much may be said in favor for a Western physician of our own Medical Schools.

The Professors in nearly all our young Western Schools are men who were educated in the East, have had all the advantages that such schools confer, besides the long years they have spent among and combating with the very diseases the Western student expects to spend his life; and unless *our* Professors are very "degenerate sons of noble sires," *ceteris paribus*, they are more competent to teach Western student than most of those who so honorably fill the chairs in the schools in the Eastern States.

Then, what we would say to the Western student, is, *exhaust the*

*sources of learning in your home Medical Schools*, and then if you have time and money to spare, visit, not only our Eastern, but the schools of England and continental Europe, where you may store your minds with all the rich treasures of science and art which the oldest and best can bestow. When returning you may bless your native land, by devoting the maturity of a widely cultivated mind to the best interests of suffering humanity.

But if you have neither time nor means for such an expenditure, your time as a general rule will be better spent in exhausting the medical sources at home, studying the diseases incidental to your climate and locality, than by seeking that at foreign schools, which in many instances is rather ornamental than useful, to the exclusion of that which will render you a useful and successful physician.

There is another point to which I wish to call the attention of our Western physicians: and that is, write for your home journals; communicate facts, every thing new or interesting, either as it may relate to articles of the materia medica, discoveries in the modus operandi of medicines in their application to given cases of pathology. Scarcely an epidemic occurs, when the intelligent scrutiny of the practitioner will not discover some new feature or peculiarity not hitherto observed. If these facts were all faithfully transmitted to the medical journal of your State or section, what a mass of clinical information would be gathered and in turn scattered broad-cast to all parts of the country, furnishing important data for the evolution of great principles which are to serve as beacon lights to guide the student of nature and the practicing physician in times of uncertainty and doubt, peering up amid the gloom of professional and scientific investigation, like a light house to the tempest tossed mariner, enabling him to guide his laboring bark in safety to the calm and peaceful haven of rest. So such facts and principles aid the physically and mentally over-tasked physician in seasons of doubt and peril, when some important case is vibrating between life and death. If the patient be the father of a large and helpless family, entirely dependant upon his exertions for support, the pleadings of that heart-stricken and suffering family, in looks more eloquent than words, 'oh, save my husband, save my father,' ring in his ears, and vibrate in his very soul. What then would he give for some principle, some fact, that should enable him to rebuke the disease, calm the throbbing heart and bid health and vigor once more course through his frame and mantle his cheek—granting him the power, under Providence, to give back the husband and father to that weeping, trembling, rejoicing family.

Then, friends, treasure up your experience, be willing to contribute your share to add to the motive power that shall move on the car of progress in professional and scientific improvement: so shall you become benefactors of your race, add to your own professional renown, and have the satisfaction of feeling at the close of life, that you have done your duty.

